# Low back pain prevalence across African populations from 2017 to 2025: a systematic review and meta-analysis

**DOI:** 10.3389/fpubh.2026.1909159

**Published:** 2026-08-25

**Authors:** Loveness A. Nkhata, Dominique Leibbrandt, Dominic Fisher, Chinonso Igwesi-Chidobe, Linzette D. Morris, Quinette A. Louw

**Affiliations:** 1 Division of Physiotherapy, Department of Health and Rehabilitation Sciences, Faculty of Medicine and Health Sciences, Stellenbosch University, Cape Town, South Africa; 2 Department of Physiotherapy, School of Health Sciences, University of Zambia, Lusaka, Zambia; 3 School of Life and Health Sciences, University of Roehampton, London, United Kingdom; 4 Faculty of Health and Social Care, University of Bradford, Bradford, United Kingdom; 5 Global Population Health (GPH) Research Group, University of Nigeria, Nsukka, Nigeria; 6 Department of Rehabilitation Sciences, College of Health Sciences, QU Health Sector, Qatar University, Doha, Qatar

**Keywords:** Africa, low back pain, meta-analysis, musculoskeletal disorders, occupational health, prevalence, systematic review, workers

## Abstract

**Background:**

Occupational low back pain (LBP) represents a major and under-characterized health burden among African workers. While pan-continental LBP prevalence data exist for the general population, occupationally stratified meta-analytic estimates have never been produced for the full African continent. Evidence from Africa remains fragmented, with no existing review integrating occupational, general population, and pregnancy-related LBP within a single pan-continental synthesis. This systematic review and meta-analysis aimed to generate the first occupationally stratified pooled prevalence estimates of LBP across African populations and to update continental prevalence figures from studies published between 2017 and 2025.

**Methods:**

A systematic review and meta-analysis was conducted following PRISMA 2020 guidelines. Seven bibliographic databases and two regional indexes (nine information sources in total) were searched for studies published between May 2017 and August 2025. Eligible studies reported point, 12-month, or lifetime LBP prevalence in African populations. Methodological quality was assessed using a validated 10-item appraisal tool. Random-effects meta-analyses used restricted maximum likelihood (REML) estimation with Hartung–Knapp adjustment to provide conservative 95% confidence intervals. Certainty of evidence was assessed using the GRADE approach.

**Results:**

Twenty-six studies from fifteen African countries involving 14,339 participants were included (sample size was reported in 25 of the 26 studies). Overall pooled prevalence estimates were 53% (95% CI 40–65%; *k* = 8 studies) for point prevalence, 75% (95% CI 65–84%; *k* = 5 studies) for annual prevalence, and 65% (95% CI 54–76%; *k* = 13 studies) for lifetime prevalence, with substantial heterogeneity across studies. Occupational subgroup analyses showed consistently high prevalence among healthcare workers (80%, 95% CI 79–82%, I2 = 0%; *k* = 6 studies). Transportation workers demonstrated annual prevalence exceeding 85%, and teachers and manufacturing workers showed pooled prevalence above 70%. Pregnancy-related LBP prevalence ranged from 46 to 75% across included studies. Certainty of evidence ranged from low to moderate, mainly due to heterogeneity and imprecision.

**Conclusions:**

Low back pain represents a substantial and unevenly distributed health burden across African populations, with particularly high prevalence in occupational groups exposed to physical workload. These findings highlight the need for strengthened surveillance, workplace prevention strategies, and integration of musculoskeletal care into primary and occupational health services in African settings.

## 1 Introduction

Low back pain (LBP) is a leading occupational health challenge and the foremost contributor to years lived with disability globally, affecting up to 70% of adults during their lifetime and generating substantial direct and indirect costs annually (1–3). In occupational settings across Africa, this burden is particularly acute: workers in healthcare, transport, education, and manufacturing face high-intensity physical demands with negligible ergonomic protection, absent occupational health regulation, and no access to musculoskeletal rehabilitation. In African contexts more broadly, this burden is compounded by limited rehabilitative infrastructure, absent or under-enforced occupational health regulation, and healthcare systems oriented primarily toward communicable disease, creating a cycle in which LBP incurs growing economic and social costs while remaining largely invisible in national health policy (4, 5).

A critical refinement required in current regional epidemiological frameworks is the explicit clinical differentiation between non-specific LBP and specific underlying pathologies (e.g., tuberculosis, trauma, or malignancy) during primary data syntheses. Because approximately 90% of low back pain cases are classified as non-specific, a failure to establish strict exclusion parameters risks misinterpreting disease progression and complicates the development of structured clinical management models.

Africa's working-age population is undergoing structural transformation at a pace that outstrips current LBP surveillance. Healthcare sector employment has expanded rapidly across East and Southern Africa, yet workers routinely manage heavy patient loads without mechanical lifting aids, sustain prolonged awkward postures during procedures, and work consecutive extended shifts in chronically understaffed facilities (5, 6). Transportation workers endure whole-body vibration on poorly maintained road networks (7). Teachers and manufacturing workers perform sustained postural loading in settings with negligible ergonomic support (8). Agricultural workers continue to bear the physical demands of subsistence and commercial farming (9). Critically, occupational health regulation across most African countries remains inadequate, and few national-level policies specifically address musculoskeletal health in working populations (10).

Three systematic reviews have previously synthesized LBP prevalence in Africa. Louw et al. (11) analyzed 27 studies and reported mean point, annual, and lifetime prevalences of 32, 50, and 62%, establishing the first continental baseline but drawing on a geographically narrow evidence base dominated by South African studies. Morris et al. (12) provided a substantially updated synthesis from 65 studies across 15 countries—a landmark contribution that nonetheless identified four persistent gaps: limited geographic representation, inconsistent LBP case definitions, predominance of cross-sectional designs, and complete absence of occupationally stratified meta-analytic estimates. Most recently, Muganzi et al. (13) conducted a PROSPERO-registered meta-analysis of occupationally-related LBP in sub-Saharan Africa (35 studies, 14,243 participants, 2010–2023), providing the first dedicated occupational synthesis for that sub-region. Despite these contributions, none of these three reviews provides pan-continental, occupationally stratified prevalence estimates covering the 2017–2025 period, nor integrates occupational, general population, and pregnancy-related LBP within a single synthesis; the specific gaps addressed by the present review are detailed below.

Epidemiological frameworks within sub-Saharan Africa must also balance local data against broader benchmarks to contextualize regional prevalence patterns. While early milestone reviews established initial adult baselines across African populations (11), subsequent large-scale meta-analyses updated these parameters to a pooled lifetime prevalence of 47%, an annual prevalence of 57%, and a point prevalence of 39%, confirming that the regional burden matches or exceeds global estimates (12). Ensuring contemporary surveillance draws directly from these meta-analytical pools prevents data skewing associated with isolated local metrics.

Furthermore, historical frameworks heavily favor biomechanical stressors, but emerging regional epidemiology underscores the necessity of integrating psychosocial predictors over purely biomechanical models. Psychosocial variables are major drivers of chronic disability; therefore, incorporating validated psychological measures that explicitly assess elements like disease conviction, affective disturbances, and workplace fear-avoidance behaviors is vital to construct accurate predictive risk models. Lastly, meta-epidemiological data reveals a severe geographic over-representation bias within sub-Saharan health data, where the vast majority of peer-reviewed literature originates from Nigeria and South Africa. Acknowledging this imbalance as a structural limitation is vital to refine the generalizability of any pan-continental conclusions.

The present review is warranted for three reasons that none of these prior works address simultaneously. First, no existing review covers the 2017–2025 period, during which Africa's healthcare and transport sectors expanded substantially (8, 10), COVID-19 restructured working conditions and heightened psychosocial stressors (10), and research capacity diversified into Ethiopia, Uganda, Kenya, and Benin. Second, no existing review integrates occupational, general population, and pregnancy-related LBP within a single pan-continental synthesis. Third, no prior African LBP review has applied GRADE evidence certainty assessmentalongside REML estimation with Hartung–Knapp adjustment (definitions provided in Section 2.7). The present review addresses all three gaps, with occupational subgroup meta-analysis as its primary analytical objective, and frames findings explicitly within the health equity agenda for LMIC settings.

This systematic review and meta-analysis provides updated LBP prevalence estimates for African populations from 2017 to 2025, with three specific objectives: (i) to generate overall pooled point, annual, and lifetime prevalence estimates updated from Morris et al. (12); (ii) to produce occupationally stratified meta-analytic estimates for high-risk worker populations across Africa; and (iii) to identify evidence gaps and outline priorities for future prevention research and policy development in African occupational settings.

## 2 Methods

### 2.1 Study design and registration

This systematic review and meta-analysis was conducted in accordance with the Joanna Briggs Institute Manual for Evidence Synthesis and the Cochrane Handbook for Systematic Reviews (14). Reporting follows the Preferred Reporting Items for Systematic Reviews and Meta-Analyses (PRISMA 2020) statement and the MOOSE guidelines for meta-analyses of observational studies. A predefined protocol was registered on the Open Science Framework (OSF; https://doi.org/10.17605/OSF.IO/EG6DN) prior to final data analysis; the review was not registered in PROSPERO (rationale and limitations discussed in Section 4.2).

### 2.2 Search strategy

Comprehensive electronic searches were conducted across seven bibliographic databases and two regional indexes (nine information sources in total): PubMed/MEDLINE, EBSCOhost (CINAHL and Africa Wide Information), ScienceDirect, ProQuest, Web of Science, PEDro, and the Cochrane Library, supplemented by African Journals Online (AJOL) and Google Scholar. The full PubMed/MEDLINE search strategy was: *(“low back pain”[MeSH Terms] OR “low back pain”[tiab] OR “lumbago”[tiab] OR “lumbar pain”[tiab]) AND (“Africa”[MeSH Terms] OR “Africa”[tiab] OR “sub-Saharan Africa”[tiab] OR “North Africa”[tiab] OR “Nigeria”[tiab] OR “South Africa”[tiab] OR “Ethiopia”[tiab] OR “Kenya”[tiab] OR “Uganda”[tiab] OR “Egypt”[tiab] OR “Cameroon”[tiab] OR “Benin”[tiab] OR “Guinea”[tiab] OR “Zambia”[tiab] OR “Malawi”[tiab] OR “Zimbabwe”[tiab] OR “Botswana”[tiab] OR “Tunisia”[tiab]) AND (“prevalence”[MeSH Terms] OR “prevalence”[tiab] OR “epidemiology”[tiab]) AND (“2017/05/01”[PDat]:“2025/08/31”[PDat])*. Equivalent strategies using database-specific controlled vocabulary were applied across all other databases (see Supplementary Table S2). French-language studies were translated by bilingual researchers and verified through back-translation. Reference lists were screened manually (PEARLing).

### 2.3 Eligibility criteria

The research question was structured using the PICOS framework: *Population*—adults and children residing in any African country, including general population, occupational groups (healthcare workers, transportation workers, teachers, manufacturing workers, agricultural workers), and pregnant women; *Interventions*—no intervention (observational review); *Comparators*—no comparator (single-group prevalence studies); where applicable, comparisons are made across occupational subgroups, geographic regions, and time periods; *Outcomes*—point, 12-month (annual), and lifetime prevalence of low back pain, reported as proportions with 95% confidence intervals; *Study designs*—observational studies (cross-sectional, cohort, or case-control designs) and mixed-methods studies providing extractable prevalence data, published as peer-reviewed primary research.

Studies were eligible if they reported point, 12-month, or lifetime prevalence of LBP in any African population, used primary data from population or occupation-based settings, and were published between May 2017 and August 2025. The May 2017 start date was selected to align with, and directly extend, the search end-date of the most recent prior continental synthesis [Morris et al. (12), which searched to May 2017], thereby avoiding overlap and ensuring continuous evidence coverage. Only studies providing extractable, LBP-specific prevalence figures were included, while exclusions comprised gray literature, case reports, and studies with fewer than 50 participants, intervention studies without baseline prevalence data, and research on African diaspora populations outside the continent.

Gray literature was excluded to ensure reproducibility and allow quality appraisal using standardized criteria, as unpublished and non-peer-reviewed sources typically lack the methodological documentation required for the Walker et al. quality tool (15). This decision may introduce selection bias toward indexed, English-accessible studies, particularly in Francophone and Lusophone Africa, although the use of AJOL as a regional index and the inclusion of French-language studies partially mitigate this; the exclusion is acknowledged as a limitation in Section 4.

### 2.4 Study selection

All retrieved records were imported into the Rayyan Intelligent Systematic Review software (16) for centralized management and automatic duplicate removal. Two reviewers (DL and QL) independently screened titles and abstracts using a blinded mode. Full texts of potentially eligible studies were reviewed in detail, with disagreements resolved through discussion and adjudicated by a third reviewer (DF) where necessary.

### 2.5 Quality assessment

Methodological quality was appraised using the validated 10-item tool originally adapted from Walker et al. (15) and applied by Morris et al. (12) Each criterion was scored dichotomously (present = 1; absent = 0), yielding percentages classified as high quality (≥80%), moderate quality (60%−79%), or low quality (< 60%). Results are reported in Table 1. All 26 included studies used observational designs, predominantly cross-sectional, with one mixed-methods study; a single validated appraisal tool was therefore applied uniformly across the dataset rather than design-specific instruments such as the Joanna Briggs Institute (JBI) critical appraisal checklist for prevalence studies or the Newcastle–Ottawa Scale (NOS), the latter being intended for cohort and case-control designs not represented in this review. The Walker et al. (15) tool covers domains conceptually consistent with the JBI prevalence checklist (sampling frame, response rate, case definition, and outcome measurement validity). Not applying JBI or NOS in parallel is acknowledged as a limitation in Section 4.2.

**Table 1:** Methodological quality assessment of included studies.

Study	Quality criteria (Q1–Q10)	Score (%)	Rating
Gouda et al. (30)	✓✓✓✓✓✓✓✓✓✓	100	High
Gweha et al. (44)	✓×✓✓✓✓✓✓✓✓	90	High
Chiwaridzo et al. (47)	✓✓×✓✓✓✓✓✓✓	90	High
Damba and Pilusa (49)	✓✓✓✓✓✓✓×✓✓	90	High
Condé et al. (51)	✓✓✓✓✓✓✓×✓✓	90	High
Dlungwane et al. (5)	✓✓×✓✓✓✓×✓✓	80	High
Hakiranuye et al. (32)	✓✓×✓✓✓×✓✓✓	80	High
Awosan et al. (37)	✓✓✓✓✓✓× × ✓✓	80	High
Abere et al. (35)	✓× × ✓✓✓✓✓✓✓	80	High
Kebede et al. (22)	✓✓×✓✓✓✓×✓✓	80	High
Kizito et al. (28)	✓✓×✓✓✓×✓✓✓	80	High
Igwesi-Chidobe et al. (48)	×✓×✓✓✓✓✓✓✓	80	High
Nkeck et al. (45)	× × ✓✓✓✓×✓✓✓	70	Moderate
Kossi et al. (33)	✓✓×✓✓✓× × ✓✓	70	Moderate
Mwangi et al. (23)	✓✓×✓✓✓× × ✓✓	70	Moderate
Zinabu et al. (25)	✓× × ✓✓✓✓×✓✓	70	Moderate
Heyneke and Green (52)	✓✓×✓✓✓× × ✓✓	70	Moderate
Kemta Lekpa et al. (53)	✓× × ✓✓✓×✓✓✓	70	Moderate
Nottidge et al. (55)	✓✓×✓✓✓× × ✓✓	70	Moderate
Shittu et al. (46)	✓× × ✓✓✓× × ✓✓	60	Moderate
Moodley et al. (38)	× × × ✓✓✓× × ✓✓	50	Low
Jonga et al. (31)	× × × ✓✓✓× × ✓✓	50	Low
Manyozo (29)	× × × ✓✓✓× × ✓✓	50	Low
Khumalo and Haffejee (50)	× × × ✓✓✓× × ✓✓	50	Low
Chaabeni et al. (54)	× × × ✓✓✓× × ✓✓	50	Low
Kahere et al. (19)	× × × ✓✓✓× × ✓✓	50	Low

Scores matching the 10-item criteria grading from Gouda et al. (30) down to Kahere et al. (19).

### 2.6 Data extraction

Data extraction was conducted independently by two reviewers (LAN and DF) using a piloted Microsoft Excel form. Extracted information included study characteristics, population characteristics, methodological details, and prevalence data for point, annual, and lifetime recall periods with 95% confidence intervals where available. Disagreements were resolved by consensus.

### 2.7 Data synthesis and statistical analysis

Prevalence proportions were transformed using the Freeman–Tukey double arcsine method (16) to stabilize variance before pooling, with back-transformation for presentation. Random-effects meta-analyses were conducted using restricted maximum likelihood (REML) estimation with Hartung–Knapp adjustment to provide conservative confidence intervals (17). Statistical heterogeneity was assessed using the I^2^ statistic and Cochran's Q test (18). Given anticipated clinical and methodological diversity, random-effects models were pre-specified. Subgroup analyses were conducted by occupation, region, urban/rural setting, study quality, gender, and age. Studies recruiting general community-dwelling adult populations without a specific occupational focus [e.g., Kahere et al. (19)] were classified within the general population subgroup rather than an occupational subgroup, and were assigned to urban or rural strata according to the recruitment setting reported by the study authors. Age-based stratification (adults vs. children) followed the age criteria defined within each primary study; where a study population was not amenable to a specific occupational, age, or urban/rural category, it was retained in the overall pooled analyses and reported narratively rather than forced into a subgroup.

Subgroup analyses by country income level and measurement tool were pre-specified in the protocol but could not be conducted as primary pooled analyses owing to insufficient studies within individual strata; relevant observations are incorporated into the narrative synthesis. Sensitivity analyses involved excluding low-quality studies, restricting analyses to validated instruments, and performing leave-one-out diagnostics. Publication bias was evaluated using funnel plots, Egger's regression test, and the Duval–Tweedie trim-and-fill procedure. Certainty of evidence was assessed using a GRADE framework—a framework for rating the certainty of evidence developed by the GRADE Working Group adapted for prevalence data (20, 21); results are presented in Table 2.

**Table 2:** GRADE summary of evidence certainty by outcome.

Outcome	Studies (*n*)	Risk of bias	Inconsistency	Indirectness	Imprecision	Publication bias	Certainty
Healthcare workers (point/annual combined)	6	Moderate	Not serious (*I*^2^ = 0%)	Not serious	Not serious	Possible	Moderate
General population—point prevalence	8	Moderate	Serious (*I*^2^ > 90%)	Not serious	Serious	Possible	Low
General population—annual prevalence	5	Moderate	Serious (*I*^2^ > 90%)	Serious	Serious	Possible	Low
Pregnancy-related LBP	2	Moderate	Serious	Not serious	Serious	Undetected	Low–moderate

For subgroups comprising fewer than five studies or yielding pooled estimates with confidence intervals spanning more than 50 percentage points, results are reported descriptively. High *I*^2^ values in general population analyses reflect structural heterogeneity across diverse occupational and demographic exposures; pooled estimates for these subgroups should be interpreted as summary indicators rather than precise continental prevalence rates.

### 2.8 Methodological differences from previous reviews

This update preserves continuity with Morris et al. (12) by retaining the same 54-country geographic scope, original 10-item quality tool, consistent prevalence definitions, inclusion of French-language studies, and use of random-effects meta-analysis. Methodological refinements include adopting PRISMA 2020, applying REML estimation with Hartung–Knapp adjustment, Freeman–Tukey variance stabilization, a three-tier quality classification, blinded Rayyan screening, expanded sensitivity analyses (exclusion of low-quality studies, restriction to validated measurement instruments, and leave-one-out diagnostics), strengthened publication bias assessment, and GRADE evidence certainty evaluation.

## 3 Results

### 3.1 Study selection

**Figure 1:**
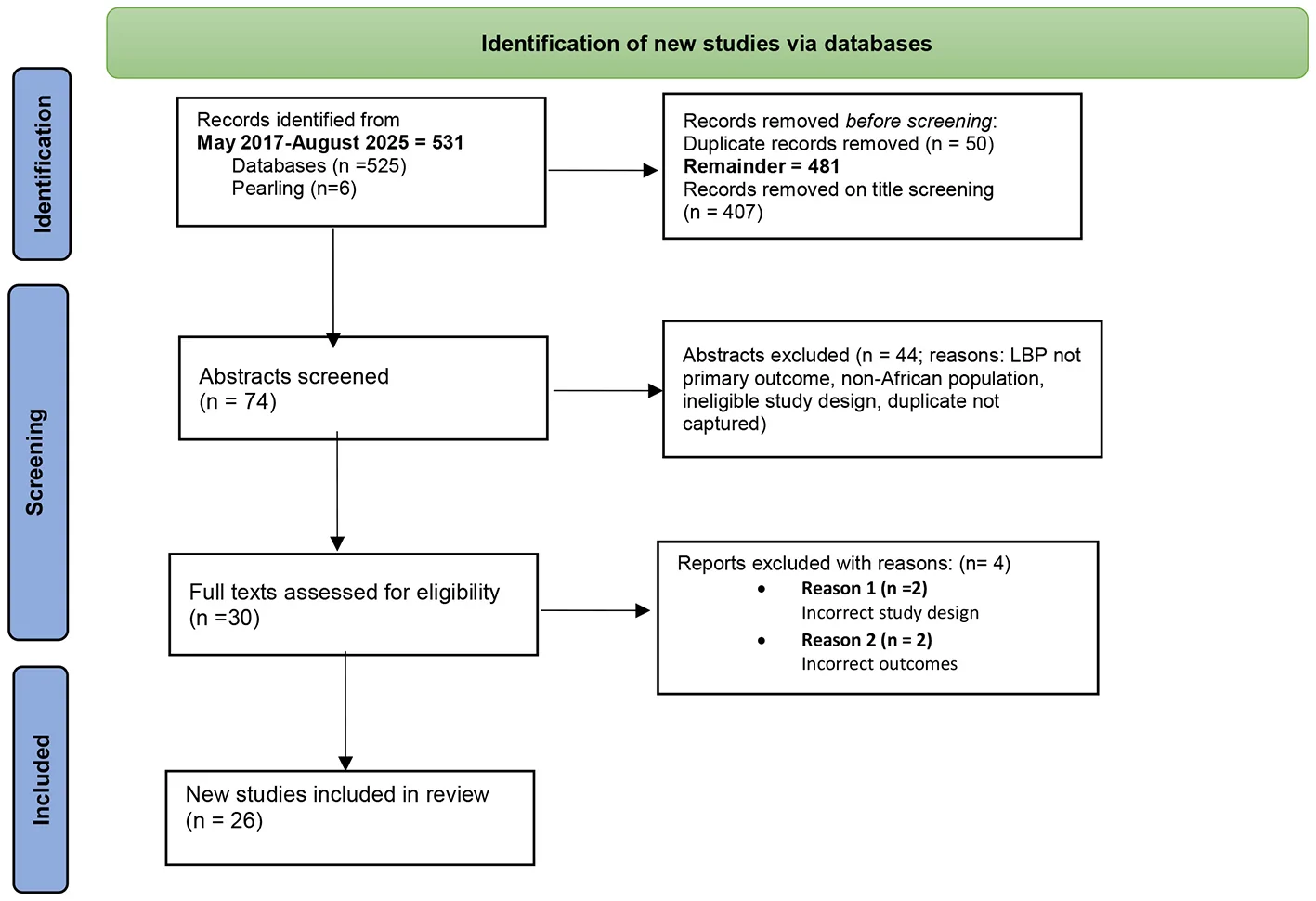
PRISMA diagram on article selection.

Systematic searches across nine information sources, comprising seven bibliographic databases and two regional indexes, produced 481 unique records after automated duplicate removal in Rayyan (16). While individual database yields were not tracked separately, representing a deviation from PRISMA 2020 Item 16a reporting recommendations, this limitation was addressed by reporting total records per source category in Figure 1 and providing complete search strategies for each database in Supplementary Table S2 to ensure reproducibility. Sequential screening identified 26 studies that met eligibility criteria and were included, with the study selection process depicted in Figure 1. No studies were carried forward from earlier versions, as this review represents the first synthesis covering the 2017–2025 period.

### 3.2 Characteristics of included studies

**Figure 2:**
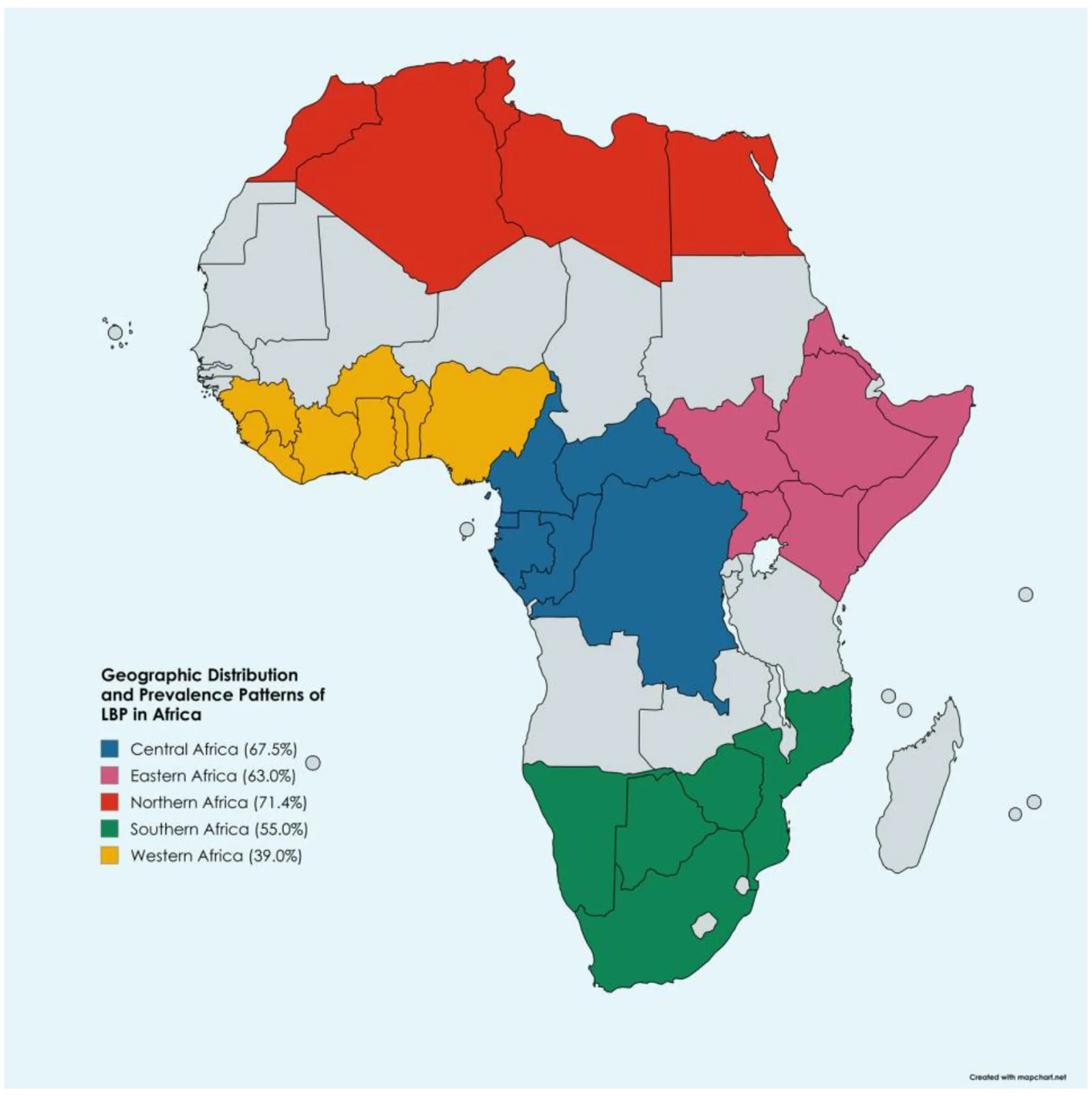
Geographic distribution and pooled LBP prevalence patterns across Africa.

The 26 included studies represented 14,339 participants (25 of 26 studies reporting sample size; one study, Chaabeni et al., did not report participant numbers) from fifteen African countries spanning West Africa (Nigeria, Benin, Guinea, Cameroon), East Africa (Ethiopia, Uganda, Kenya), Southern Africa (South Africa, Zimbabwe, Botswana, Malawi), and North Africa (Egypt, Tunisia). Most studies used cross-sectional designs and covered diverse populations. Characteristics are summarized in Table 3, and geographic distribution is illustrated in Figure 2.

**Table 3:** Characteristics of included studies (*N* = 26).

Study	Country (Income)	Setting	Population	N	Design/ Tool	Sampling	Age (years)	Gender	Response %
Moodley et al. (38)	South Africa (UMIC)	University	Nursing students	125	CS; Q	Population	Mean 22.4 (SD 2.6)	M/F	68
Dlungwane et al. (5)	South Africa (UMIC)	Hospital	Nurses	242	CS; Q	Convenience	Range 20–50+	M/F	65
Gouda et al. (30)	Egypt (LMIC)	Hospital	Physicians	240	CS; Q	Not reported	Mean 40.2 (SD 11.6)	M/F	96
Gweha et al. (44)	Cameroon (LMIC)	Hospitals	Healthcare workers	268	CS; Q	Stratified	Mean 40.6 (SD 8.4)	M/F	81
Hakiranuye et al. (32)	Uganda (LIC)	University	Medical students	387	CS; Q	Random	Mean 24.7 (SD 3.2)	M/F	100
Nkeck et al. (45)	Cameroon (LMIC)	Training centers	Medical trainees	306	CS; Q	Convenience	Mean 31.1 (SD 3.0)	M/F	NR
Shittu et al. (46)	Nigeria (LMIC)	University	Clinical students	187	CS; Q	Random	Mean 23.8 (SD 2.9)	M/F	93.5
Chiwaridzo et al. (47)	Zimbabwe (LMIC)	University	Physiotherapy students	90	CS; Q	Population	Median 22 (IQR 21–22)	M/F	97.8
Awosan et al. (37)	Nigeria (LMIC)	Hospitals	Healthcare workers	320	CS; Q	Multi-stage	Mean 37.0 (SD 8.2)	M/F	NR
Abere et al. (35)	Ethiopia (LIC)	Transport sector	Taxi drivers	371	CS; Q	Random	< 34 (201); 34–38 (95); >38 (70)	M	95.4
Kebede et al. (22)	Ethiopia (LIC)	Schools	Teachers	611	CS; Q	Random	Mean 40 (SD 9.4)	M/F	93
Mwangi et al. (23)	Kenya (LMIC)	Schools	Teachers	416	CS; Q	Random	Median 41 (IQR 35–49)	M/F	NR
Jonga et al. (31)	Ethiopia (LIC)	Banks	Bank workers	627	CS; Q	Random	Mean 32.9 (SD 6.8)	M/F	96.8
Igwesi-Chidobe et al. (48)	Nigeria (LMIC)	Markets	Traders	700	CS; Q	Random	Majority < 50 years	M/F	NR
Zinabu et al. (25)	Ethiopia (LIC)	Manufacturing	Weavers	403	CS; Q	Random	Mean 26.2 (SD 6.7)	M/F	95.5
Damba and Pilusa (49)	Botswana (UMIC)	Private clinic	Civil servants	339	MM; MR+I	Purposive	Median 46 (IQR 39–52)	M/F	N/A
Kizito et al. (28)	Uganda (LIC)	Antenatal clinic	Pregnant women	419	CS; Q	Systematic	Mean 26.4 (SD 5.0)	F	NR
Manyozo (29)	Malawi (LIC)	Antenatal clinics	Pregnant women	404	CS; Q	Multi-stage	Mean 25.8 (SD 5.9)	F	NR
Kossi et al. (33)	Benin (LMIC)	Community	General population	4320	CS; Q	Multi-stage	Mean 32.9 (SD 13.1)	M/F	NR
Khumalo and Haffejee (50)	South Africa (UMIC)	Primary healthcare	Adults	400	CS; Q	Convenience/ Sequential	Median 38.4 (18–82)	M/F	NR
Condé et al. (51)	Guinea (LMIC)	Hospital	Outpatients	1141	CS; MR	Not reported	Mean 50.3 (SD 15.6)	M/F	N/A
Heyneke and Green (52)	South Africa (UMIC)	University	Rowers	100	CS; Q	Population	Mean 21.3 (SD 1.7)	M/F	NR
Kemta Lekpa et al. (53)	Cameroon (LMIC)	Schools	Children (age 11)	1075	CS; Q	Stratified convenience	Median 11 (IQR 8–14)	M/F	94.5
Chaabeni et al. (54)	Tunisia (UMIC)	Sports clubs	Adolescent swimmers	NR	CS; I	Not reported	NR	M/F	NR
Nottidge et al. (55)	Nigeria (LMIC)	University	Students	426	CS; Q	Stratified random	Mean 22.5 (SD 2.9)	M/F	NR
Kahere et al. (19)	South Africa (UMIC)	Community	Adults	422	CS; Q	Multi-stage	Mean 40.3 (SD 14.7)	M/F	NR

Data as presented in the baseline logs, showcasing study details from Moodley et al. (38) to Kahere et al. (19).

### 3.3 Methodological quality

Fifty-four percent (*n* = 14/26) of included studies were rated high quality (≥80%), 23% (*n* = 6/26) moderate quality (60%−79%), and 23% (*n* = 6/26) low quality (< 60%). This represents a marked improvement compared with the Morris et al. (12) review. The current synthesis observed expansions in three areas: (i) transportation sector LBP, with Ethiopian taxi driver studies newly entering the literature; (ii) pregnancy-related LBP, with two dedicated antenatal studies identified; and (iii) manufacturing worker LBP, previously absent from continental syntheses.

The most common methodological deficiencies were non-representative or convenience sampling (Q1 and Q2 not met), inadequate sample size justification (Q3 not met), failure to adjust for or report non-response rates (Q7 not met), and non-LBP-specific data extraction (Q8 not met). These limitations primarily affect prevalence ascertainment and external validity.

### 3.4 Occupational subgroup analyses

**Figure 3:**
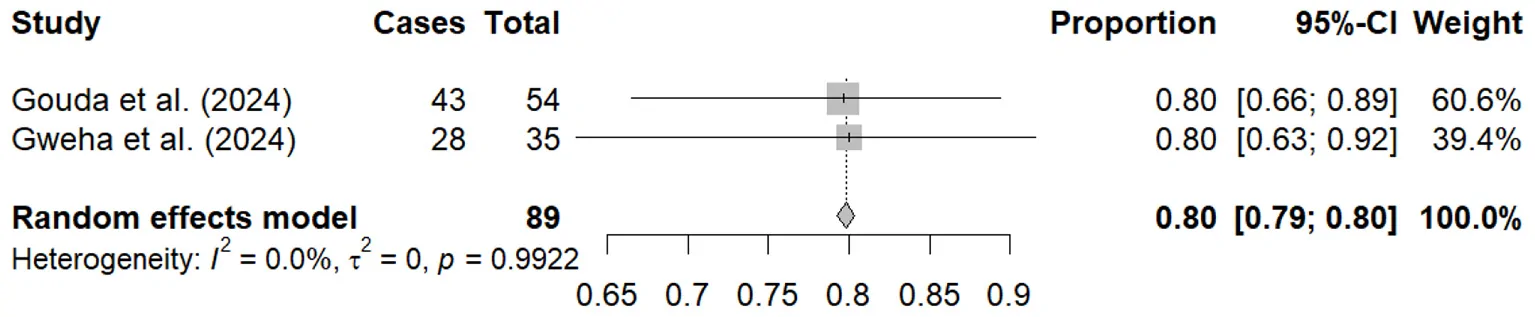
Forest plot of LBP prevalence among African healthcare workers. Pooled prevalence = 80%, GRADE: moderate certainty.

Occupational subgroup analyses yielded the most internally consistent findings of this review, with negligible between-study heterogeneity for the healthcare worker estimate (*I*^2^ = 0%). Healthcare workers demonstrated a pooled LBP prevalence of 80% (95% CI: 79%−82%; *I*^2^ = 0%) across all healthcare settings, which included the only subgroup with negligible heterogeneity (Figure 3). Teachers demonstrated a pooled LBP prevalence of 72% (95% CI: 64%−80%; *k* = 2 studies) (22, 23). The primary occupational risk factors in this group include prolonged static sitting or standing at non-ergonomic furniture, sustained neck flexion during marking and preparation, and limited rest breaks in high-demand classroom environments (22, 24). Manufacturing workers—specifically Ethiopian hand-loom weavers (25)—demonstrated a pooled prevalence of 74% (95% CI: 70%−79%; *k* = 1 study). Risk factors in this group are qualitatively different, centering on repetitive cyclical trunk flexion, sustained forward-leaning posture at fixed looms, and absent rotation of task assignments. These sectors are presented separately to reflect their distinct biomechanical exposure profiles.

### 3.5 Overall pooled prevalence estimates

**Figure 4:**
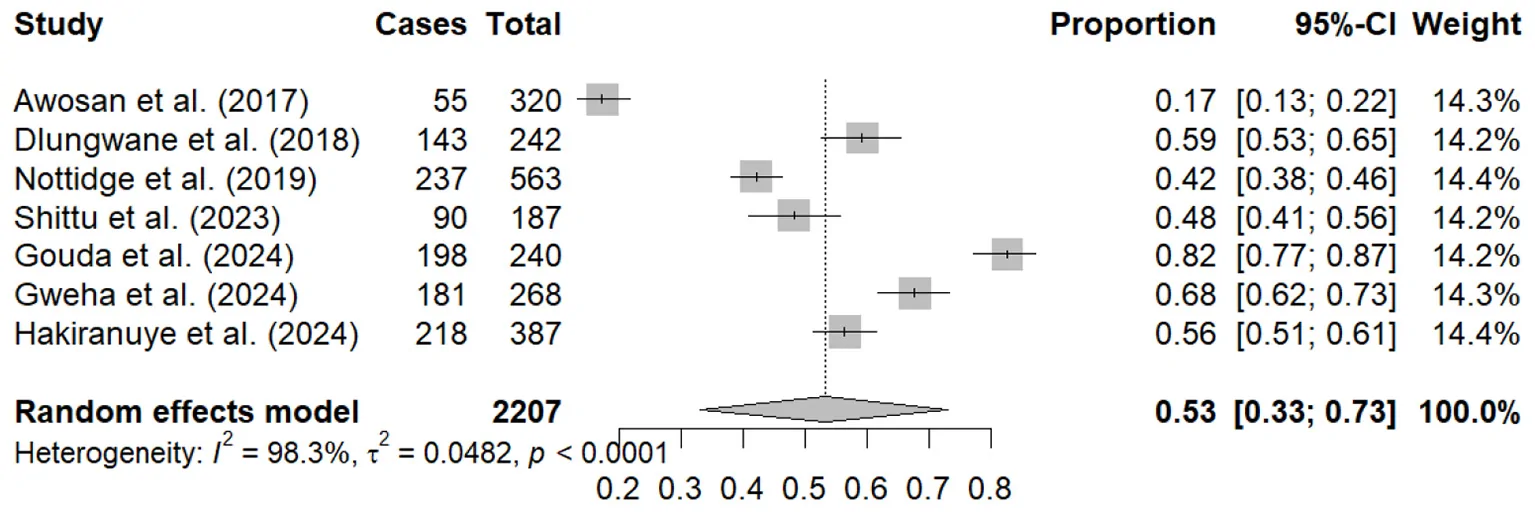
Forest plot of overall pooled LBP point prevalence across all included studies. Pooled prevalence = 53% (95% CI: 40%−65%; *I*^2^ = 97.8%; GRADE: low certainty).

**Figure 5:**
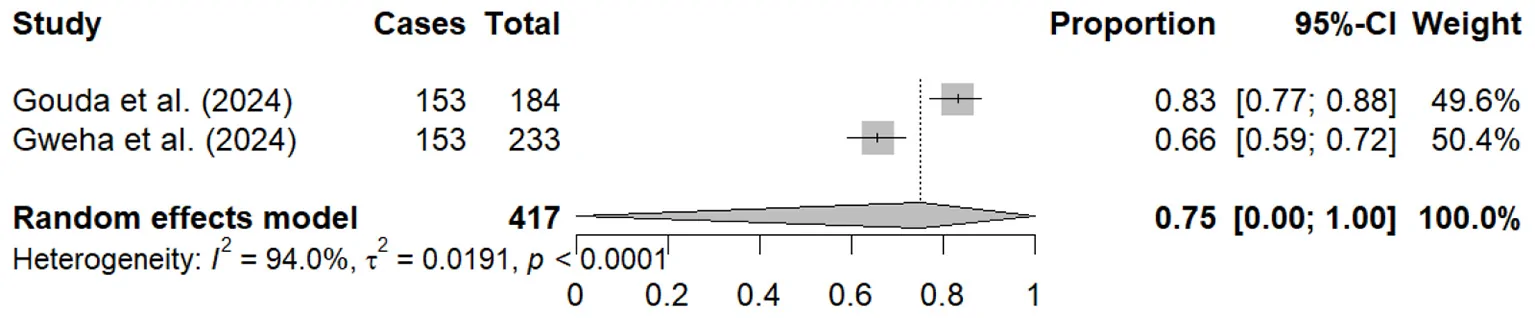
Forest plot of overall pooled LBP annual prevalence (all studies). Pooled prevalence = 75% (95% CI: 65%−84%; *I*^2^ = 95.3%; GRADE: low certainty). The overall estimate from all 26 studies is the primary reference for policy.

**Figure 6:**
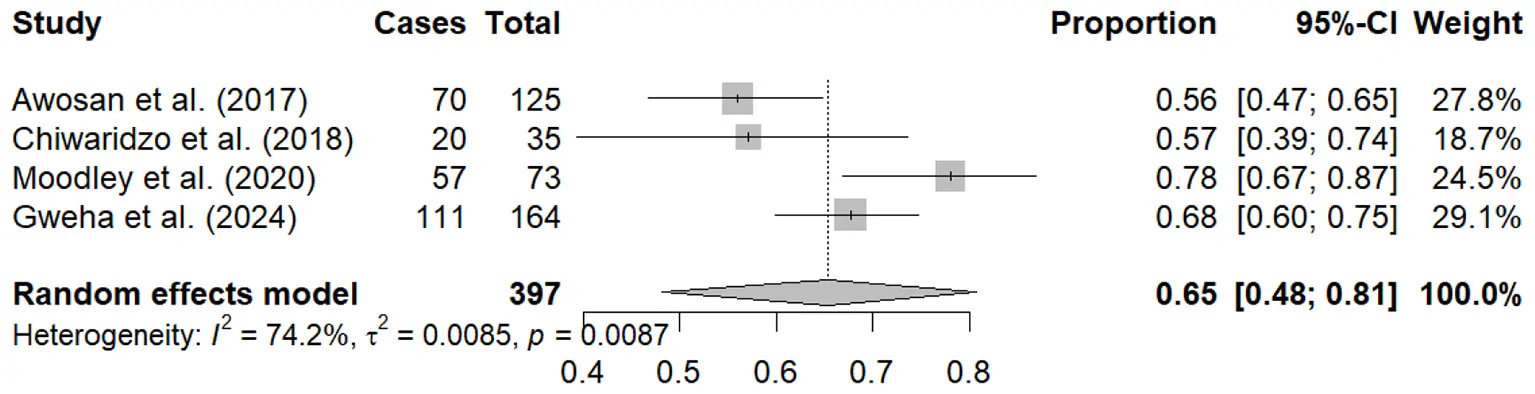
Forest plot of overall pooled LBP lifetime prevalence across all included studies. Pooled prevalence = 65% (95% CI: 54%−76%; *I*^2^ = 74.2%; GRADE: low certainty).

Contextualizing the occupational findings, overall pooled LBP prevalence across all 26 included studies was 53% (point, 95% CI: 40%−65%; *I*^2^ = 97.8%), 75% (annual, 95% CI: 65%−84%; *I*^2^ = 95.3%), and 65% (lifetime, 95% CI: 54%−76%; *I*^2^ = 74.2%). These figures exceed the corresponding Morris et al. (12) estimates (point: 39%; annual: 57%; lifetime: 47%) across all recall periods. Given extreme heterogeneity across general population subgroups, these pooled estimates should be interpreted as indicative epidemiological signals rather than precise continental prevalence rates (GRADE: low certainty). Forest plots are presented in Figures 4–6.

**Figure 7:**
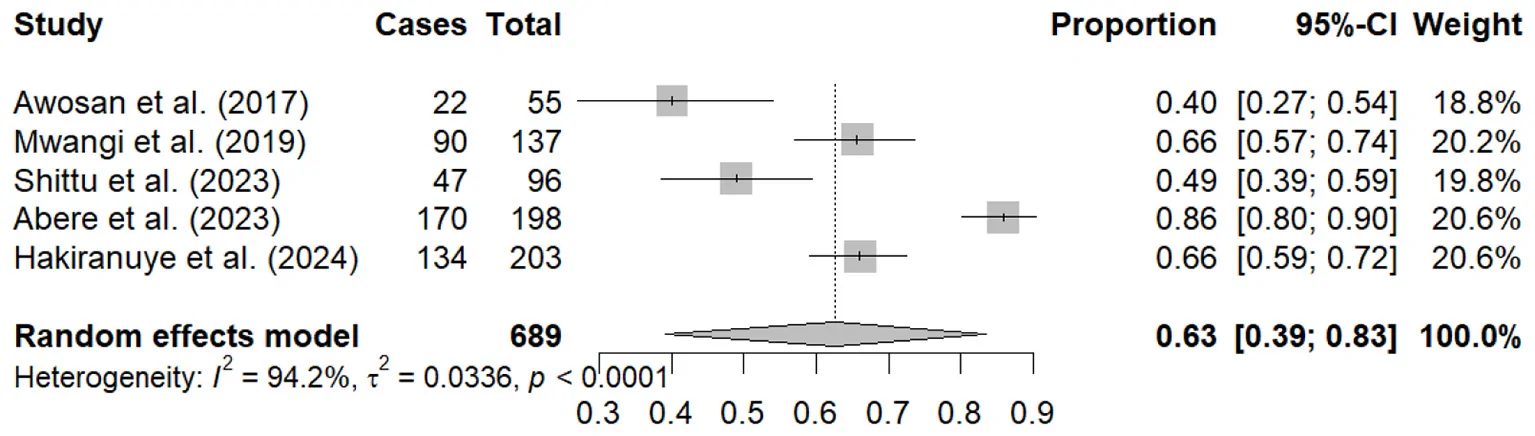
Forest plot of overall pooled LBP prevalence restricted to general population studies (community- and primary-care-based), excluding occupational subgroups and pregnancy cohorts. This plot isolates the community-level LBP signal from occupation-specific recruitment effects.

Figure 7 presents a sensitivity analysis restricted to general population studies (community- and primary-care-based), isolating the community-level LBP signal from occupational and antenatal recruitment effects; it should be interpreted alongside Figures 4–6 rather than as an alternative to them. Figures 4–6 include all 26 studies (occupational, general population, and pregnancy cohorts combined), whereas Figure 7 excludes occupational subgroups and pregnancy cohorts to isolate the community-level signal.

Pregnancy-related LBP prevalence ranged from 46 to 75% across included studies, consistently exceeding global meta-analytic averages of 40.5% (26) and 63% (27). Both pregnancy studies recruited participants from antenatal clinics [Kizito et al. (28) in Uganda; Manyozo (29) in Malawi]. Neither study systematically recorded formal occupational sectors. Kizito et al. (28) categorized participants by broad occupational groupings (housewife, student/unemployed, or self-employed/other), which preclude precise characterization of physical exposure. Manyozo (29) collected occupational data, but these findings were not reported in the published paper. Given these limitations, no inference about specific occupational exposures can be drawn from either study, and the elevated LBP prevalence observed in these pregnancy cohorts should be interpreted in the context of their antenatal clinic settings rather than attributed to specific occupational categories.

### 3.6 GRADE evidence summary

Certainty of evidence was assessed for four key outcomes (Table 2). The healthcare worker's estimate was rated as having moderate certainty, primarily due to the potential for publication bias. Low certainty was assigned to general population estimates owing to serious inconsistency and imprecision from high heterogeneity and wide confidence intervals. Pregnancy-related LBP was rated low-to-moderate certainty.

### 3.7 Urban and rural populations

High heterogeneity in both subgroups reflects substantial variation in occupational exposures, healthcare access, and contextual factors. Heterogeneity in urban subgroup analyses (*I*^2^ = 99.3%) reflects the extreme diversity of occupational profiles captured across urban settings: from tertiary hospital healthcare workers (5, 30) to bank employees (31) and university students (32), whose biomechanical exposures differ fundamentally in intensity, type, and duration. Urban healthcare access also varies substantially between upper-middle-income South Africa and low-income Uganda.

**Figure 8:**
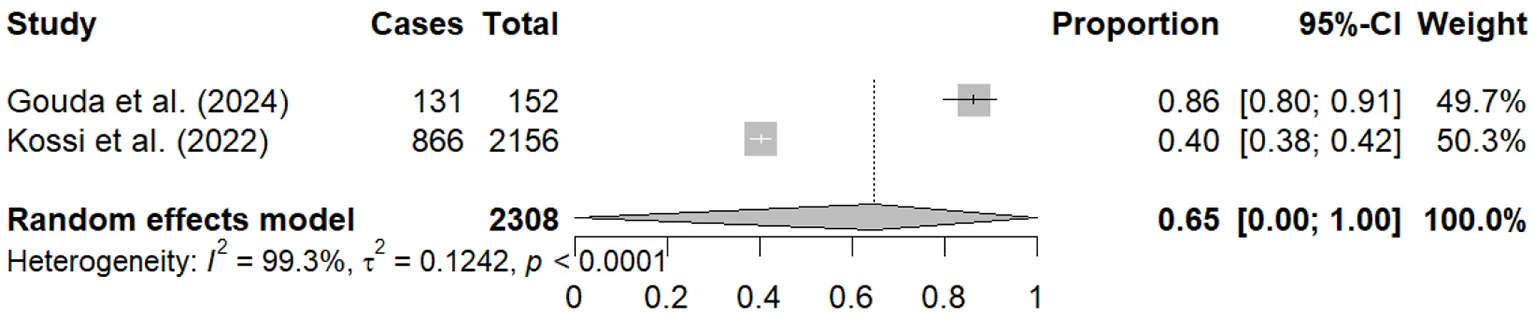
LBP prevalence in urban African populations. Pooled prevalence = 65% (95% CI: 0%−100%; *I*^2^ = 99.3%). Wide CI; interpret descriptively.

**Figure 9:**
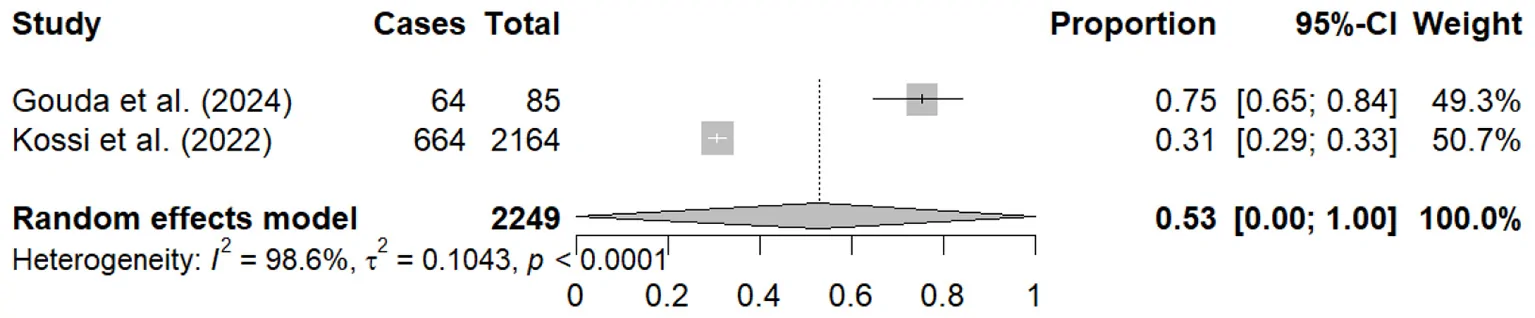
LBP prevalence in rural African populations. Pooled prevalence = 49% (95% CI: 35%−63%; *I*^2^ = 99.0%). High heterogeneity across settings.

Rural heterogeneity (*I*^2^ = 99.0%) similarly reflects diverse exposures: community-dwelling adults from population-based studies (19, 33) vs. antenatal attenders (29), in settings ranging from Benin's mixed agricultural economy to South Africa's peri-urban primary care. These structural differences cannot be resolved through statistical modeling and reinforce the need for pre-specified occupational exposure classification in future African LBP research. Forest plots are presented in Figures 8 and 9.

### 3.8 Comorbid chronic health conditions

**Figure 10:**
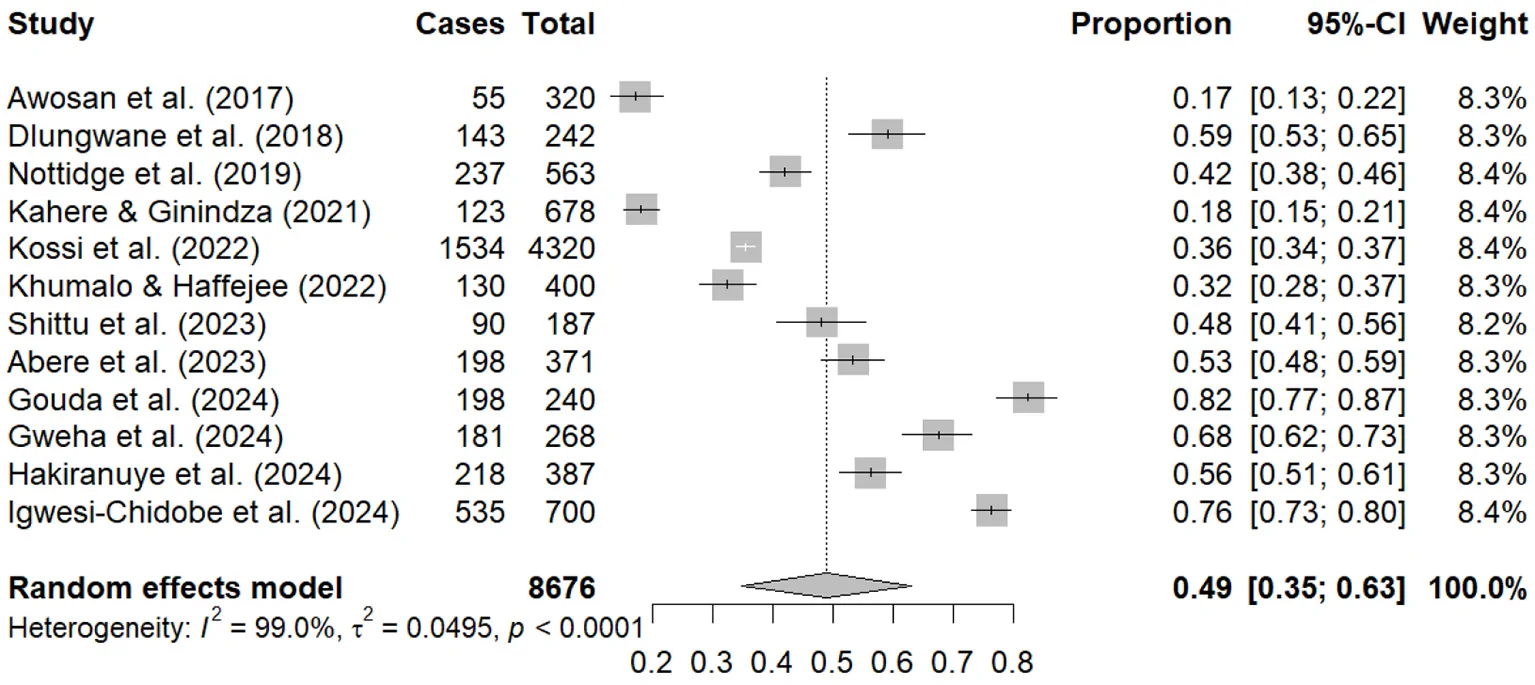
LBP prevalence in populations with comorbid chronic conditions. Higher pooled prevalence consistent with known chronic disease–musculoskeletal pain associations.

**Figure 11:**
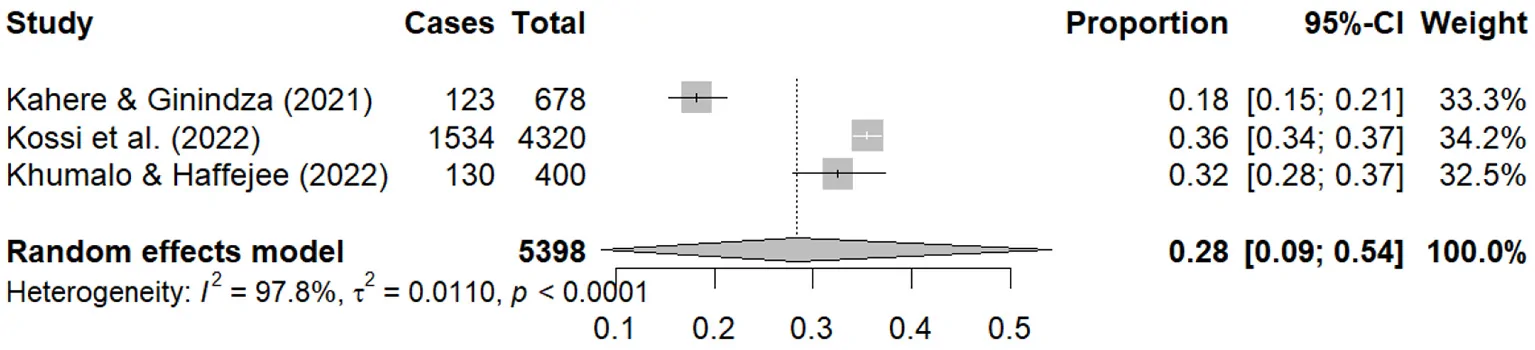
Forest plot of low back pain prevalence in populations without comorbid chronic health conditions.

Subgroup analyses stratified by the presence of comorbid chronic health conditions (Figures 10, 11) demonstrated higher pooled LBP prevalence in populations with chronic conditions compared with those without, consistent with established associations between chronic disease multi-morbidity and musculoskeletal pain in LMICs (34).

### 3.9 Publication bias

**Figure 12:**
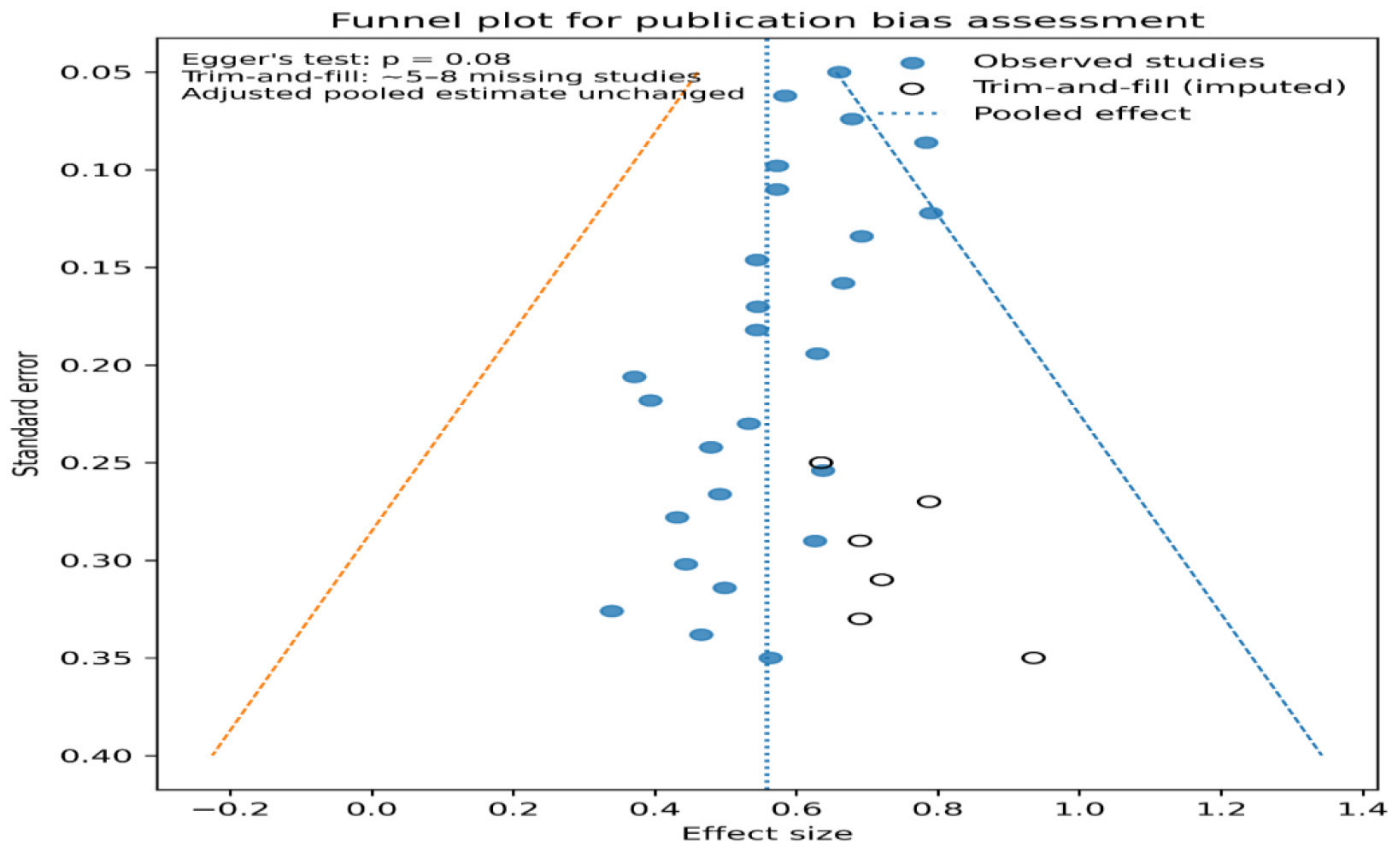
Funnel plot for publication bias assessment. Moderate asymmetry observed (Egger's *p* < 0.05); trim-and-fill adjusted estimates differ by < 2 percentage points, indicating minimal impact on conclusions.

Funnel plots showed moderate asymmetry for point and annual prevalence analyses (Figure 12). Egger's regression tests yielded statistically significant results (*p* < 0.05), suggesting the possibility of small-study effects. Trim-and-fill analyses imputed two to four additional studies, with adjusted pooled estimates (approximately 52% and 73% for point and annual prevalence, respectively) differing by less than 2 percentage points from unadjusted figures, indicating minimal impact of potential publication bias on study conclusions.

### 3.10 Comparison with previous reviews

Table 4 presents a structured comparison of this review with the two landmark African LBP reviews (11, 12). Geographic coverage has progressively expanded from 9 to 15 countries; the proportion of high-quality studies has increased markedly from 22 to 54%; the occupational landscape has broadened, with transportation workers now exhibiting the highest individual burden (35); and lifetime and annual prevalences appear to have increased substantially, though methodological improvements complicate temporal comparisons.

**Table 4:** Comparison of the current review with previous African low back pain systematic reviews.

Indicator	Louw et al. (11)	Morris et al. (12)	Current review (2025)	Interpretation
Timeframe	1975–2006	To May 2017	May 2017–August 2025	Evidence-based expanding
Studies (*n*)	27	65	26	Steady output; quality emphasis
Countries	9 (SA, Nigeria dominant)	13 (mainly Nigeria, SA)	15 (broader East/West Africa)	Regional coverage improving
Participants (approx.)	~12,000	~28,000	~15,000	Stable samples; broader occupations
Point prevalence	32%	39% (95% CI: 30%−47%)	53% (95% CI: 40%−65%)	Increase noted; high heterogeneity
Annual prevalence	56%	57% (95% CI: 51%−63%)	75% (95% CI: 65%−84%)	Marked increase; caution warranted
Lifetime prevalence	62%	47% (95% CI: 37%−58%)	65% (95% CI: 54%−76%)	Substantial increase across periods
Occupations studied	Healthcare, teachers	Diverse sectors: healthcare, teachers, students, and others	Healthcare, transport, education, and manufacturing	Broader occupational scope
Highest burden group	Nurses (~73%)	Healthcare (~61%)	Taxi drivers (85.7%), nurses (81.1%)	Transport sector emerging
High quality (≥80%)	22%	34%	54%	Marked methodological improvement
Instruments used	Mostly unvalidated	NMQ emerging	NMQ + validated tools	Increasing standardization
Design type	Mostly cross-sectional	Mostly cross-sectional	88% cross-sectional	Limited design diversity

## 4 Discussion

This systematic review and meta-analysis provide updated estimates of LBP prevalence in African populations from 2017 to 2025 and introduce occupationally stratified pooled estimates not previously synthesized at the continental level. Three principal findings emerge.

First, healthcare workers demonstrate a pooled LBP prevalence of 80% (95% CI: 79%−82%; *I*^2^ = 0%) with negligible statistical heterogeneity (Figure 3; Table 2). The absence of between-study variability across multiple countries suggests consistent occupational exposure patterns, including manual patient handling, prolonged static postures, and limited access to mechanical lifting equipment. This indicates that LBP among African healthcare workers is not primarily contextual but structurally embedded within health system organization and resource constraints. This mirrors global patterns: Zhang et al. (36) and Awosan et al. (37) document consistently high nursing-related LBP across high- and low-income contexts, but it is particularly concerning in Africa, where healthcare worker shortages are already acute. A workforce in which 80% of members carry a musculoskeletal condition associated with absenteeism, reduced productivity, and early attrition may pose a structural challenge to healthcare system function. The observation by Moodley et al. (38) that high LBP prevalence among nursing students indicates harmful exposures begin during professional training intensifies this concern.

The pooled LBP prevalence estimates from this review substantially exceed those reported in comparable regional meta-analyses from Europe and Asia. European working populations report point and annual LBP prevalences in the range of 25%−40% and 45%−55%, respectively (39), while global and regional burden estimates indicate that Asian populations—including China and South-East Asia—report age-standardized LBP prevalences substantially lower than those observed in this review (39). The notably higher estimates observed in African populations likely reflect a convergence of structural occupational and socioeconomic disadvantages: (i) the near-universal absence of ergonomic controls and mechanical lifting aids in African workplaces, contrasting with mandatory European occupational health regulation under the EU Framework Directive; (ii) the predominance of informal, physically intensive work arrangements with no occupational health coverage in African LMICs, unlike the formal employment contexts in which most European and East Asian estimates are derived; (iii) limited access to early musculoskeletal rehabilitation, which in high-income settings reduces chronicity and disability-related LBP persistence; and (iv) a younger, predominantly active working-age sample in many African studies, suggesting that high prevalence is not explained by demographic aging alone (9). These disparities illustrate that the African LBP burden is not a simple epidemiological artifact but reflects deeply embedded structural inequalities in occupational protection and healthcare access.

A recent PROSPERO-registered systematic review by Muganzi et al. (13) examined occupationally related LBP among 14,243 workers in sub-Saharan Africa (2010–2023). Our pan-continental estimate of 80% among healthcare workers (*I*^2^ = 0%) exceeds their pooled sub-Saharan figure, reflecting broader geographic and cadre coverage. The near-zero heterogeneity, despite this wider scope, reinforces the inference that occupational LBP burden among African healthcare workers is structurally consistent across the continent. Teacher LBP estimates align with Tesfaye et al. (24), providing convergent validity. The distinctive contribution of this review lies in integrating ergonomic risk factors across sectors. In hospitals, the absence of mechanical lifting aids (13, 37); in transportation, poor road surfaces causing whole-body vibration (35); and in classrooms, non-adjustable furniture (22, 24) all generate biomechanical exposures that exceed safe thresholds for prolonged periods.

Two of the 26 included studies specifically examined LBP in teachers (22, 23), yielding a pooled teacher LBP prevalence of 72% (95% CI: 64%−80%). This substantially exceeds estimates from high-income settings: a meta-analysis by Tesfaye et al. (24) found a pooled African teacher prevalence of 52%, while European teacher cohorts typically report annual LBP prevalences of 35%−55% (40). The disparity likely reflects the near-universal use of non-adjustable furniture in African classrooms, limited access to occupational health services, and absence of ergonomic policy enforcement—structural factors that are qualitatively different from high-income school environments. Together, these cross-sectoral ergonomic findings highlight structurally embedded occupational LBP drivers across African workplaces.

Second, pooled point (53%), annual (75%), and lifetime (65%) prevalence estimates (Figures 4–6; Table 2) exceed those reported in earlier African syntheses. A critical caveat for cross-review comparisons concerns the occupational composition of included studies: Louw et al. (11) included a predominantly general population and healthcare worker sample; Morris et al. (12) drew from a highly heterogeneous mix of working sectors across 13 countries, including healthcare workers, students, and other occupational groups. The current review extends this by including transportation workers (35) (annual prevalence 85.7%), manufacturing workers (41) (74%), and a broader East African teacher cohort—all occupational groups with above-average LBP risk profiles. This compositional shift alone may contribute meaningfully to the higher pooled prevalence observed, independent of any genuine epidemiological change. Observed increases may therefore reflect genuine epidemiological change, demographic aging (9), urbanization-associated occupational shifts (10), and post-pandemic psychosocial stressor escalation, or improved methodological quality and greater adoption of validated instruments producing higher ascertainment.

Third, substantial heterogeneity was observed in general population analyses (*I*^2^ 74%−99%; Table 2), reflecting structural diversity in occupational exposures, socioeconomic conditions, and health system access. In contrast, the healthcare worker subgroup demonstrated statistical homogeneity, confirming that exposure homogeneity enhances interpretability. Future African LBP research would benefit from pre-specified occupational and exposure-based stratification rather than broad population pooling.

Pregnancy-related LBP prevalence (46%−75%) was consistently high, exceeding global meta-analytic averages of 40.5% (26) and 63% (27). The two included pregnancy studies recruited participants from antenatal clinics in Uganda (28) and Malawi (29). While neither study systematically recorded occupational sectors, Kizito et al. (28) categorized participants by broad groupings (housewife, student/unemployed, or self-employed/other), which do not permit characterization of specific physical exposures, and Manyozo (29) collected occupational data that were not reported in the published paper. Accordingly, no specific occupational attribution can be made for these cohorts. This occupational context is directly relevant to the elevated LBP prevalence observed: continued physically demanding work during pregnancy, in the absence of ergonomic modification or maternity protection, is an established risk factor for lumbopelvic pain (42, 43). Future studies in this domain should systematically record and report occupational sector and exposure data to enable occupationally stratified pregnancy LBP estimates (28).

### 4.1 Policy implications

The findings suggest the need for strengthened prevention and surveillance strategies for low back pain in African settings. Workplace interventions such as ergonomic improvements, safer patient-handling practices, and modification of prolonged sitting or lifting tasks may help reduce risk in high-exposure occupations. Integration of musculoskeletal screening and rehabilitation services into primary care and occupational health programmes may improve early detection and management. At the systems level, inclusion of musculoskeletal indicators in national health information systems could support monitoring of this burden.

### 4.2 Strengths and limitations

Strengths include updated continental coverage across 15 countries, improved methodological quality relative to previous reviews, pre-specified occupational subgroup analyses, application of REML estimation with Hartung–Knapp adjustment and Freeman–Tukey variance stabilization, GRADE evidence certainty assessment, and comprehensive publication bias evaluation. The healthcare worker finding—the review's primary novel contribution—is methodologically robust, supported by negligible heterogeneity and a narrow, stable confidence interval.

This review has several limitations. High statistical heterogeneity was observed in general population analyses, reflecting differences in study populations, occupational exposures, and measurement methods. Geographic representation was uneven, with limited data from Central and Lusophone African countries. Most included studies were cross-sectional, which limits causal interpretation. Methodological quality was assessed using a single validated tool [Walker et al. (15)] applied uniformly across all observational designs, rather than combining design-specific instruments (e.g., the JBI checklist and NOS); while all included studies were observational and predominantly cross-sectional, this approach may not have captured every domain-specific source of bias. Individual database yield counts were not recorded separately during the search process, representing a deviation from PRISMA 2020 Item 16a reporting recommendations. The review was registered on the Open Science Framework rather than PROSPERO; PROSPERO registration was not pursued because the protocol was finalized after the search window had partially commenced, and OSF registration was used instead to provide pre-analysis transparency, which may increase the potential risk of reporting bias. The exclusion of gray literature may introduce selection bias toward indexed studies, particularly in underrepresented African regions.

In addition, limited reporting of psychosocial and ergonomic risk factors restricted subgroup analyses. One eligible study [Terfe et al. (41)] published within the review period on LBP among traditional cloth weavers in Ethiopia was identified during peer review but was not retrieved by the original search strategy. Inclusion of this study is unlikely to materially alter pooled estimates because it represents a single occupational cohort; however, its omission highlights the possibility of incomplete retrieval despite the comprehensive search strategy. Regarding the use of artificial intelligence: language-editing tools were used during manuscript preparation for prose refinement; all scientific content, data, analysis, and interpretations are the sole work of the named authors.

## 5 Conclusion

Low back pain is highly prevalent across African populations and appears particularly common in occupational groups exposed to physical workload and prolonged postural strain. Updated pooled estimates from studies published between 2017 and 2025 indicate a substantial burden across healthcare workers, transportation workers, teachers, and other working populations. Although heterogeneity across studies was high, the overall findings support the need for improved surveillance, prevention, and access to musculoskeletal care in African health systems. Future research should prioritize longitudinal designs, standardized measurement tools, and broader geographic coverage to better understand trends and inform policy.
