# Supplementary material for: Low back pain prevalence across African populations from 2017 to 2025: a systematic review and meta-analysis
Source: Front Public Health. 2026 Aug 25;14:1909159. doi: 10.3389/fpubh.2026.1909159 (PMC13552109; doi:10.3389/fpubh.2026.1909159)
Supplement: Supplementary file 1 [file Data_Sheet_1.pdf]

Supplementary Table S1: GRADE Evidence Certainty Assessment for All Outcomes

LBP Prevalence across African Populations — Systematic Review & Meta-Analysis (2017–2025)

GRADE domains: Risk of Bias, Inconsistency, Indirectness, Imprecision, Publication Bias. Starting certainty: High. Each serious concern downgrades by one level; very serious by two.

Outcome / Subgroup	n	Risk of Bias	Inconsistency (I^2)	Indirectness	Imprecision	Publication Bias	GRADE Certainty	Notes / Key Findings
**A. OCCUPATIONAL SUBGROUPS**								
**Healthcare workers (point + annual combined)**	6	Moderate (cross-sectional designs)	Not serious (I^2 = 0%)	Not serious	Not serious (CI: 79%–82%)	Possible (Egger p < 0.05)	**MODERATE** ⊕⊕⊕◯	Primary finding. Pooled prevalence 80% (95% CI: 79%–82%). Only subgroup with negligible heterogeneity. Downgraded once for publication bias only.
**Transportation workers (annual)**	3	Moderate	Serious (I^2 > 85%)	Not serious	Serious (wide CI)	Undetected	**LOW** ⊕⊕◯◯	Annual prevalence >85%. Ethiopian taxi drivers dominant. Wide CI; reported descriptively (†).
**Teachers (point prevalence)**	3	Moderate	Serious (I^2 > 80%)	Not serious	Serious (wide CI)	Undetected	**LOW** ⊕⊕◯◯	Pooled prevalence >70%. Kenya and Ethiopia contributors. Reported descriptively (†).
**Manufacturing / industrial workers**	2	Moderate	Serious	Not serious	Very serious (only 2 studies)	Undetected	**VERY LOW** ⊕◯◯◯	Insufficient studies for reliable pooled estimate; reported descriptively.
**Medical students / clinical trainees**	3	Moderate	Serious (I^2 > 75%)	Moderate (student populations)	Serious	Undetected	**VERY LOW** ⊕◯◯◯	Emerging burden; high heterogeneity across settings.
**B. OVERALL POOLED ESTIMATES**								
**All studies — point prevalence**	26	Moderate	Serious (I^2 = 97.8%)	Not serious	Serious (CI: 40%–65%)	Possible	**LOW** ⊕⊕◯◯	Pooled 53% (95% CI: 40%–65%). Extreme heterogeneity; indicative signal. Exceeds Morris et al. (39%).
**All studies — annual prevalence**	18	Moderate	Serious (I^2 = 95.3%)	Not serious	Serious (CI: 65%–84%)	Possible	**LOW** ⊕⊕◯◯	Pooled 75% (95% CI: 65%–84%). Exceeds Morris et al. (57%).
**All studies — lifetime prevalence**	14	Moderate	Moderate (I^2 = 74.2%)	Not serious	Moderate (CI: 54%–76%)	Undetected	**LOW** ⊕⊕◯◯	Pooled 65% (95% CI: 54%–76%). Lower heterogeneity. Exceeds Morris et al. (47%).
**C. GEOGRAPHIC REGION**								
**East Africa**	12	Moderate	Serious (I^2 > 90%)	Not serious	Serious	Possible	**LOW** ⊕⊕◯◯	Ethiopia (n=5), Kenya (n=3), Uganda (n=2), Tanzania (n=1), Rwanda (n=1). Largest regional bloc.
**West Africa**	7	Moderate	Serious (I^2 > 85%)	Not serious	Serious	Undetected	**LOW** ⊕⊕◯◯	Nigeria (n=4), Ghana (n=2), Senegal (n=1).
**Southern Africa**	5	Moderate	Moderate (I^2 60%–80%)	Not serious	Moderate	Undetected	**LOW** ⊕⊕◯◯	South Africa dominant (n=3); Malawi (n=1); Zimbabwe (n=1).
**Central / North Africa**	2	Moderate	Serious	Serious (limited countries)	Very serious	Undetected	**VERY LOW** ⊕◯◯◯	Underrepresented region; Cameroon only LMIC contributor. Descriptive only.
**D. SETTING (URBAN / RURAL)**								

**Urban populations**	6	Moderate	Serious (I² = 99.3%)	Not serious	Very serious (CI: 0%–100%)	Possible	**VERY LOW** ⊕◯◯◯	Pooled 65% but CI spans full range; interpret descriptively only.
**Rural populations**	5	Moderate	Serious (I² = 99.0%)	Not serious	Serious (CI: 35%–63%)	Undetected	**LOW** ⊕⊕◯◯	Pooled 49% (95% CI: 35%–63%). High heterogeneity.
**E. COUNTRY INCOME LEVEL**								
**Lower-middle-income countries (LMICs)**	18	Moderate	Serious (I² > 90%)	Not serious	Serious	Possible	**LOW** ⊕⊕◯◯	Majority of included studies.
**Low-income countries (LICs)**	6	Moderate	Serious (I² > 85%)	Not serious	Serious	Undetected	**LOW** ⊕⊕◯◯	Uganda, Ethiopia, Malawi contributors.
**Non-classified / upper-middle income**	2	Moderate	Serious	Moderate	Very serious	Undetected	**VERY LOW** ⊕◯◯◯	Insufficient studies; descriptive only.
**F. SPECIAL POPULATIONS**								
**Pregnancy-related LBP**	2	Moderate	Serious (2 studies only)	Not serious	Serious (range 46%–75%)	Undetected	**LOW–MODERATE** ⊕⊕◯◯	Range 46%–75%; consistently exceeds global averages (40.5%–63%). Insufficient studies for meta-analysis.
**G. COMORBID CONDITIONS**								
**With comorbid chronic conditions**	4	Moderate	Serious (I² > 85%)	Not serious	Serious	Undetected	**VERY LOW** ⊕◯◯◯	Higher prevalence than reference; consistent with known associations.
**Without comorbid chronic conditions**	4	Moderate	Serious (I² > 80%)	Not serious	Serious	Undetected	**VERY LOW** ⊕◯◯◯	Comparator subgroup.

GRADE Certainty Symbols: ⊕⊕⊕⊕ High | ⊕⊕⊕◯ Moderate | ⊕⊕◯◯ Low | ⊕◯◯◯ Very Low: Colour coding: Yellow = Moderate | Orange = Low | Red = Very Low: † Subgroups with <5 studies or CI width >50 percentage points reported descriptively per pre-specified protocol.

Supplementary Table 2: Full Subgroup and Sensitivity Analysis Results

LBP Prevalence across African Populations — Systematic Review & Meta-Analysis (2017–2025)

Random-effects models (REML + Hartung-Knapp adjustment + Freeman-Tukey transformation). Subgroups with <5 studies or CI width >50 pp are reported descriptively (†).

Subgroup / Analysis	Prevalence Type	n Studies	Participants	Pooled Prevalence (%)	95% CI (%)	I² (%)	GRADE Certainty	Notes
**A. OCCUPATIONAL SUBGROUPS**								
**Healthcare workers (point + annual combined)**	Point + Annual	6	>2,500	80%	79%–82%	0%	**MODERATE**	Primary novel finding. Consistent across all healthcare settings. Only subgroup with negligible heterogeneity.
**Transportation workers (taxi drivers — annual)**	Annual	3	~900	>85%	† wide CI	>85%	**LOW**	† Reported descriptively. Ethiopian taxi drivers dominant. Prolonged sitting + whole-body vibration.
**Teachers**	Point	3	~1,100	>70%	† wide CI	>80%	**LOW**	† Kenya and Ethiopia contributors. Reported descriptively.
**Manufacturing / industrial workers**	Point	2	~800	NR	†	—	**VERY LOW**	† <5 studies; descriptive only.
**Medical students / clinical trainees**	Point	3	~780	>60%	† wide CI	>75%	**VERY LOW**	† Student populations; high heterogeneity.
**Bank workers**	Point	1	627	NR	—	—	**VERY LOW**	Single study (Jonga et al., 2023, Ethiopia); no pooling possible.
**B. OVERALL POOLED ESTIMATES**								
**All studies — point prevalence**	Point	26	>15,000	53%	40%–65%	97.8%	**LOW**	Exceeds Morris et al. (39%). Extreme heterogeneity; interpret as indicative epidemiological signal.
**All studies — annual prevalence**	Annual	18	>9,000	75%	65%–84%	95.3%	**LOW**	Exceeds Morris et al. (57%).
**All studies — lifetime prevalence**	Lifetime	14	>7,500	65%	54%–76%	74.2%	**LOW**	Exceeds Morris et al. (47%). Lower heterogeneity than point/annual.
**C. GEOGRAPHIC REGION**								
**East Africa**	Point + Annual	12	>7,000	~65%	† wide CI	>90%	**LOW**	Ethiopia (n=5), Kenya (n=3), Uganda (n=2), Tanzania (n=1), Rwanda (n=1).
**West Africa**	Point	7	>3,500	~55%	† wide CI	>85%	**LOW**	Nigeria (n=4), Ghana (n=2), Senegal (n=1).
**Southern Africa**	Point	5	>2,000	~60%	† wide CI	60%–80%	**LOW**	South Africa dominant (n=3); Malawi (n=1); Zimbabwe (n=1).
**Central / North Africa**	Point	2	<600	NR	†	—	**VERY LOW**	Cameroon only; underrepresented region; descriptive only.
**D. COUNTRY INCOME LEVEL**								
**Lower-middle-income countries (LMICs)**	Point + Annual	18	>10,000	~56%	† wide CI	>90%	**LOW**	Majority of included studies.
**Low-income countries (LICs)**	Point	6	>3,000	~62%	† wide CI	>85%	**LOW**	Uganda, Ethiopia, Malawi contributors.
**Non-classified / upper-middle income**	Point	2	<500	NR	†	—	**VERY LOW**	Insufficient studies; descriptive only.
**E. URBAN / RURAL SETTING**								

**Urban populations**	Point	6	>3,500	65%	0%–100%	99.3%	**VERY LOW**	Exceptionally wide CI; interpret descriptively only.
**Rural populations**	Point	5	>2,500	49%	35%–63%	99.0%	**LOW**	High heterogeneity; pooled estimate is indicative only.
**F. STUDY QUALITY (SENSITIVITY)**								
**High-quality studies only (score >=80%)**	Point	14	>8,000	~55%	† wide CI	>90%	**LOW**	Sensitivity analysis; main conclusions unchanged.
**Excluding low-quality studies (<60%)**	Point	22	>13,500	~54%	† wide CI	>90%	**LOW**	Confirms robustness of main findings.
**G. COMORBID CHRONIC CONDITIONS**								
**With comorbid chronic conditions**	Point	4	>1,500	Higher than reference	† wide CI	>85%	**VERY LOW**	Consistent with chronic disease-musculoskeletal pain associations.
**Without comorbid chronic conditions**	Point	4	>1,500	Reference	† wide CI	>80%	**VERY LOW**	Comparator subgroup.
**H. SPECIAL POPULATIONS**								
**Pregnancy-related LBP**	Point	2	~300	46%–75%	Range only	Serious	**LOW–MODERATE**	Range reported; consistently exceeds global averages (40.5%–63%). Insufficient studies for meta-analysis.
**I. SENSITIVITY ANALYSES**								
**Leave-one-out analysis — healthcare workers**	Point + Annual	6 (-1)	—	79%–81%	Stable	0%	**MODERATE**	Exclusion of any single study leaves pooled estimate essentially unchanged. Confirms robustness.
**Validated instruments only (REML sensitivity)**	Point	15	>8,000	~54%	† wide CI	>90%	**LOW**	Marginally reduced heterogeneity; conclusions unchanged.
**Trim-and-fill (publication bias correction)**	Point + Annual	26	—	Differs <2 pp	—	—	**LOW**	Adjusted estimates differ <2 percentage points from observed; publication bias has minimal effect.

† Subgroup comprises <5 studies or CI width >50 percentage points; results reported descriptively per pre-specified protocol: NR = not reported. pp = percentage points. LMICs = lower-middle-income countries; LICs = low-income countries. : GRADE certainty colour coding: Yellow = Moderate | Orange = Low | Red = Very Low | Peach = Low–Moderate.

Supplementary File S2: MOOSE Checklist

Meta-analysis of Observational Studies in Epidemiology

Stroup DF, et al. Meta-analysis of observational studies in epidemiology: a proposal for reporting. JAMA. 2000; 283(15):2008–2012. PMID: 10789670

Manuscript: Low back pain prevalence across African populations — a systematic review and meta-analysis (2017–2025) update

Nkhata LA, Leibbrandt D, Fisher D, Igwesi-Chidobe C, Morris LD, Louw QA

PLOS Global Public Health — Manuscript ID: PGPH-D-26-01140

Note: Cells highlighted in yellow have been updated to align with the revised manuscript.

Item	Description	Where Reported / Response
**Background**		
1	Problem definition	Section 1 (Introduction): Low back pain (LBP) defined as pain in the lumbar region. Prevalence among African populations identified as the primary research problem, with emphasis on occupational burden and the gap in continental evidence synthesis from 2017 onwards.
2	Hypothesis statement	Section 1, final paragraph: No formal a priori hypothesis was tested. Three specific objectives were stated: (i) generate updated pooled point, annual, and lifetime prevalence estimates for African populations (2017–2025); (ii) produce occupationally stratified meta-analytic estimates for high-risk worker groups; and (iii) identify evidence gaps and generate a policy agenda for occupational LBP prevention.
3	Description of study outcome(s)	Section 2.3 (Eligibility Criteria): Primary outcomes are point, annual (12-month), and lifetime LBP prevalence. Only studies providing extractable, LBP-specific prevalence figures were included.
4	Type of exposure or intervention used	Section 2.3: Observational review; no intervention. Exposures of interest include occupational type (healthcare, transport, teaching, manufacturing, agriculture), demographic factors (gender, age), and geographic/setting variables.
5	Type of study design used	Section 2.3: Cross-sectional prevalence studies were the predominant design. Longitudinal designs were eligible if extractable point, annual, or lifetime prevalence data were reported.
6	Study population	Section 2.3: African populations of all ages and occupational groups (general population, healthcare workers, transportation workers, teachers, manufacturing workers, pregnant women) from studies published May 2017–August 2025 across all 54 African countries.
**Search Strategy**		
7	Qualifications of searchers (e.g. librarians and investigators)	Section 2.1 & 2.2: Searches were conducted by the investigative team in accordance with the Joanna Briggs Institute Manual for Evidence Synthesis and the Cochrane Handbook for Systematic Reviews, using predefined search strings developed and verified across all nine databases.

8	Search strategy, including time period included in the synthesis and keywords	Section 2.2: Searches conducted in PubMed/MEDLINE, EBSCOhost (CINAHL and Africa Wide Information), ScienceDirect, ProQuest, Web of Science, PEDro, Cochrane Library, AJOL, and Google Scholar. Date range: May 2017–August 2025. Core search terms: 'low back pain', 'lumbago', 'lumbar pain', 'Africa', 'prevalence', 'epidemiology', and country-specific African terms. Full PubMed/MEDLINE strategy reproduced verbatim in Section 2.2; equivalent strategies in Supplementary Table S2.
9	Effort to include all available studies, including contact with authors	Section 2.2: Nine electronic databases and two regional indices searched comprehensively. Grey literature accessed via Google Scholar and AJOL. Reference lists of included studies manually screened (PEARLing). A predefined protocol was registered on the Open Science Framework (OSF; https://doi.org/10.17605/OSF.IO/EG6DN) prior to final data analysis to reduce selective reporting. No direct author contact was conducted to retrieve unpublished data.
10	Databases and registries searched	Section 2.2: PubMed/MEDLINE, EBSCOhost (CINAHL and Africa Wide Information), ScienceDirect, ProQuest, Web of Science, PEDro, Cochrane Library, African Journals Online (AJOL), and Google Scholar (nine sources total).
11	Search software used, name and version, if appropriate	Section 2.2 & 2.4: Rayyan Intelligent Systematic Review software used for centralised record management, automatic duplicate removal, and blinded screening. Standard database interfaces used for database searches. Protocol pre-registered on OSF (https://doi.org/10.17605/OSF.IO/EG6DN).
12	Use of hand searching (e.g. reference lists of obtained articles)	Section 2.2: Reference lists of all included studies were screened manually (PEARLing method) to identify additional eligible studies not captured in database searches.
13	List of citations located and those excluded, including justification	Figure 1 (PRISMA 2020 flow diagram): 481 unique records identified after automated duplicate removal; sequential screening yielded 26 included studies. Reasons for exclusion at full-text stage documented in the PRISMA diagram.
14	Method of addressing articles published in languages other than English	Section 2.2 & 2.3: No language restriction applied. French-language studies were included; translations performed by bilingual researchers and verified through back-translation. No other non-English studies were identified in the search results.
**Methods**		
15	Description of relevance or appropriateness of studies assembled for assessing the hypothesis to be tested	Section 2.3: Inclusion criteria directly aligned with review objectives — studies had to report point, 12-month, or lifetime LBP prevalence using primary data from population- or occupation-based African settings. Studies with fewer than 50 participants, grey literature, case reports, and intervention studies without baseline prevalence were excluded to ensure methodological appropriateness.
16	Rationale for the selection and coding of data (e.g. sound clinical principles or convenience)	Section 2.6: Data extracted on study design, setting, country, population type, occupation, prevalence period (point/annual/lifetime), sample size, LBP case definition, measurement tool, and quality score. Extraction form piloted before full use. Categories selected to enable subgroup analyses pre-specified in the OSF protocol.
17	Documentation of how data were classified and coded (e.g. multiple raters, blinding, and interrater reliability)	Section 2.5 & 2.6: Two independent reviewers (LAN and DF) extracted data using a piloted Microsoft Excel form; disagreements resolved by consensus. Quality appraisal conducted independently using the validated 10-item Walker et al. tool; dichotomous scoring with three-tier classification (high ≥80%, moderate 60–79%, low <60%). Results presented in Table 2.

18	Assessment of confounding (e.g. comparability of cases and controls in an observational study)	Section 2.5, 2.7 & 3.4–3.9: Potential confounding addressed through pre-specified subgroup analyses by occupation, geographic region, urban/rural setting, study quality, gender, and age. High heterogeneity in general population analyses (I^2>75%) acknowledged as reflecting structural differences in occupational and demographic exposures rather than true confounding.
19	Assessment of statistical heterogeneity	Section 2.7: I^2 statistic and Cochran's Q test used to quantify heterogeneity for all pooled analyses. Random-effects models were pre-specified in the OSF protocol given anticipated clinical and methodological diversity across African settings.
20	Description of statistical methods (e.g. complete description of fixed or random effects model, justification of whether the chosen statistical model accounts for predictors of effect size)	Section 2.7: Prevalence proportions transformed using the Freeman–Tukey double arcsine method to stabilise variance, with back-transformation for presentation. Random-effects meta-analyses used REML estimation with Hartung–Knapp adjustment for conservative confidence intervals. I^2 and Cochran's Q reported per analysis. Sensitivity analyses: exclusion of low-quality studies, restriction to validated instruments, leave-one-out diagnostics.
21	Provision of appropriate tables and graphics	Tables 1–4 and Figures 1–12: Table 1 (study characteristics), Table 2 (quality scores), Table 3 (subgroup summary), Table 4 (GRADE summary); Figure 1 (PRISMA flow), Figure 2 (geographic map), Figures 3–11 (forest plots by prevalence period and subgroup), Figure 12 (funnel plot).
**Results**		
22	Graphic summarising individual study estimates and overall estimate	Figures 3–11: Forest plots for point, annual, and lifetime prevalence and all occupational and demographic subgroups, showing individual study estimates with 95% CIs and pooled estimates. Figure 12: Funnel plot for publication bias assessment across all included studies.
23	Table giving descriptive information for each study included	Table 1: Full characteristics of all 26 included studies — country, study design, setting (urban/rural), population type, occupation, sample size, mean age, sex distribution, LBP case definition, measurement tool, and quality rating.
24	Results of sensitivity testing (e.g. subgroup analysis)	Sections 3.4–3.9: Subgroup analyses by occupation (healthcare workers, transportation, teaching, manufacturing, agriculture), geographic region, urban/rural setting, study quality, gender, age group, and comorbid conditions. Sensitivity analyses included exclusion of low-quality studies, restriction to validated measurement instruments, and leave-one-out diagnostics. Subgroups with fewer than five studies or CI spans >50 percentage points reported narratively.
25	Indication of statistical uncertainty of findings	All pooled estimates presented with 95% confidence intervals (using Hartung–Knapp adjustment) and I^2 values throughout Section 3. GRADE certainty of evidence (low to moderate) reported in Section 3 and Supplementary Table S3.
**Discussion**		
26	Quantitative assessment of bias (e.g. publication bias)	Section 3.7 & Figure 12: Funnel plot constructed for all included studies. Egger's regression test yielded a statistically significant result ($p < 0.05$), indicating asymmetry. Duval–Tweedie trim-and-fill procedure applied; adjusted pooled estimates differed by less than 2 percentage points from unadjusted estimates, indicating that potential publication bias does not materially alter conclusions.

27	Justification for exclusion (e.g. exclusion of non-English language citations)	Section 2.3 & Figure 1: All exclusion criteria documented — grey literature, case reports, <50 participants, intervention studies without baseline prevalence, African diaspora outside continent. Non-English studies were not excluded. Full exclusion reasons at full-text stage documented in PRISMA flow diagram (Figure 1).
28	Assessment of quality of included studies	Section 2.5 & Table 2: Validated 10-item quality appraisal tool (adapted from Walker et al., applied by Morris et al. 2018) used for all 26 included studies. Dichotomous scoring (present = 1, absent = 0); three-tier classification applied. 54% (14/26) rated high quality; remainder rated moderate. Quality used as a subgroup variable in sensitivity analyses.
**Conclusion**		
29	Consideration of alternative explanations for observed results	Section 4 (Discussion): Higher pooled estimates compared with prior African reviews discussed in terms of: temporal trends in Africa's healthcare and transport sector expansion post-2017; improved measurement quality in more recent studies; COVID-19-related changes in occupational conditions; and structural heterogeneity across diverse occupational exposures and demographic profiles included in general population analyses.
30	Generalisation of the conclusions (i.e. appropriate for the data presented and within the domain of the literature review)	Sections 4 & 5 (Discussion and Conclusions): Conclusions explicitly limited to African settings with acknowledgement of geographic gaps (limited Central and Lusophone African data). Context-specific policy recommendations made for LMIC settings. Generalisability caveats noted regarding cross-sectional design dominance and heterogeneity.
31	Guidelines for future research	Section 4 (Discussion) and Section 5 (Conclusions): Recommendations include standardised case definitions and measurement tools, longitudinal surveillance designs, pre-specified occupational and exposure-based stratification, expanded geographic coverage to underrepresented regions, and integration of musculoskeletal screening into primary and occupational health services across African settings.
32	Disclosure of funding source	Declarations section of manuscript: No specific funding was received for this systematic review and meta-analysis. The review was conducted as part of the academic activities of the contributing institutions.

Note: Items are reported in accordance with MOOSE 2000 (Stroup et al., JAMA 2000). Section references correspond to the submitted manuscript. OSF protocol registration: https://doi.org/10.17605/OSF.IO/EG6DN.
